# Supplementary figures and images for: CED-5/CED-12 (DOCK/ELMO) can promote and inhibit F-actin formation via distinct motifs that may target different GTPases
Source: PLoS Genet. 2024 Jul 31;20(7):e1011330. doi: 10.1371/journal.pgen.1011330 (PMC11290852; doi:10.1371/journal.pgen.1011330)

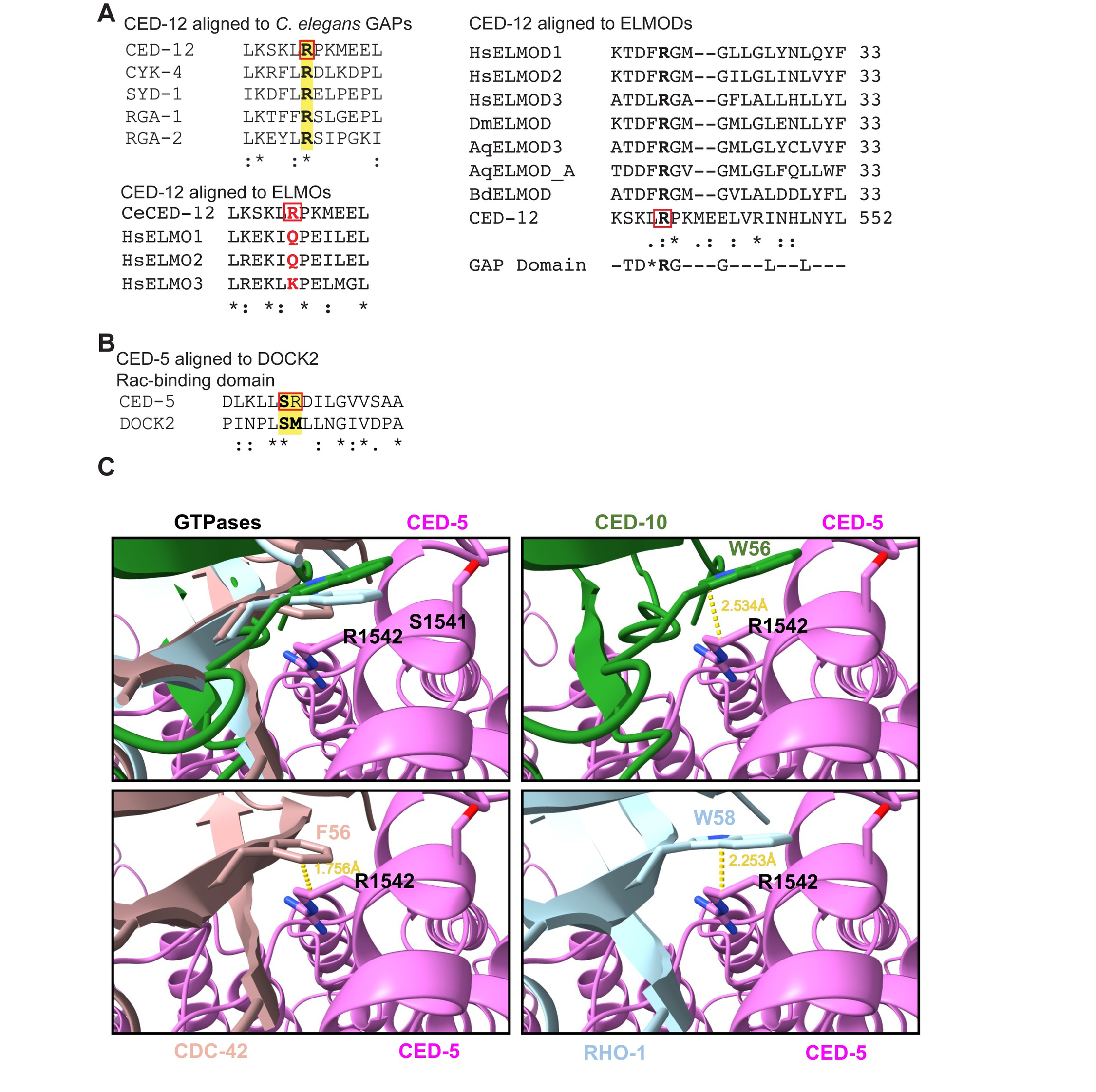

Supplement: S1 Fig — (A) Aligning CED-12 to the C. elegans GAPs CYK-4, SYD-1, RGA-1 and RGA-2 (top) identified a GAP region in CED-12. The catalytic arginine of the GAPs and CED-12 is shown in boldface and highlighted. Aligning CED-12 with human ELMOs shows a similar GAP-like region exists in ELMOs. Aligning CED-12 with ELMODs from Hs (human), Dm (Drosophila), Aq (Amphimedon queenslandica) or Bd (Batrachochytrium dendrobatidis) (bottom) illustrated they have a conserved region, that includes the arginine residue R537, boxed in red in all alignments. (B). CED-5 aligned to DOCK2 identified a conserved Rac1-binding domain. The SR mutated to AA in ced-5(pj81) is indicated. (C). DOCKs are thought to bind to Rac1 or CDC-42, but not RHO-1/RhoA. Models compare how Rac1/CED-10 W56 vs. CDC-42 F56 vs. RHO-1 W58 fit into the pocket of CED-5 S1541/R1542. See also Fig 5. (TIF) [file pgen.1011330.s001.tif]
