# Supplementary material for: CED-5/CED-12 (DOCK/ELMO) can promote and inhibit F-actin formation via distinct motifs that may target different GTPases
Source: PLoS Genet. 2024 Jul 31;20(7):e1011330. doi: 10.1371/journal.pgen.1011330 (PMC11290852; doi:10.1371/journal.pgen.1011330)
Supplement: Attachment — Submitted filename: Response to Reviewers.pdf [file pgen.1011330.s005.pdf]

March 29, 2024

Dear Drs. Chisholm and Copenhaver,

Thanks to you and the Reviewers for the suggestions for improvement. It has taken a while to make the revisions since the first author has left the lab to go to graduate school. During her winter break she added updates, especially to Figure 4, the modeling figure, now Figure 5 and Supplemental Fig. 1, which include all the revisions suggested by Reviewers. I hired and trained a new technician to help with requested revisions. She has been added as an author (Yeshaswi Pulijala).

As suggested, we have toned down the “strength of the claims,” starting with the Title and Abstract, and have revised the Figures and text in response to many of the suggestions.

To summarize the main changes to Figures:

-Figure 1A has new cartoons to introduce the phenotypes. Fig. 1 labeling was adjusted to better show the changes in mutants. Some of Fig. 1 is now in a new figure, new Fig. 2.

-We added a figure, now Figure 2, to help readers see what is meant by protrusions and retractions, with new protrusion measurements. Figure 2 shows additional evidence that F-actin regulators are elevated in the *ced-5* and *ced-12* loss of function mutants.

-Figure 3 (now Figure 4): A more representative cell corpse was added for *ced-5*. The contrast between F-actin levels in the epidermal cells as they migrate vs. within these epidermal cells as they engulf corpses is also illustrated in a new panel added to this figure, Fig. 4D.

-Figure 4 (now Figure 5), structural models: We added a Supplemental Figure to show that CDC-42 and RHO-1 cannot easily replace Rac1 in the model, as requested by one Reviewer (Suppl. Fig. 1C). Since this agrees with the work of structural biologists who were already cited (Reviewed in Nawrotek et al., 2023), we added emphasis on the published work that strongly suggests Rac1 and definitely not RHO-1 and probably not CDC-42 fit the GEF cleft of CED-5/DOCK (page 13). Revised Fig.4 (now Fig. 5) also adds cartoons to illustrate the region of ELMOs and ELMODs that align with the proposed GAP domain or CED-12 (Fig. 5A). The alignments in Suppl. Fig. 1C also include CED-12 vs. ELMOs.

-Also for Figure 4 (now 5), we made labeling and figure changes to help people better understand the structures. We labeled the DHR1 domain, the one that is predicted to stay bound to the membrane in both open and closed conformations. This should help people stay oriented relative to the membrane. We added a “membrane” to help people view the orientation of the open and closed complexes relative to membranes. We are indebted to the wonderful review by Nawrotek, Dubois, Zeghouf and Cherfils, 2023 doi:10.1002/1873-3468.14585), that echoes our interpretation of the structural data. Thus we show DOCK/ELMO bound to a curved membrane in the open conformation.

-Flipping Figure 5 and 6 (now 6 and 7): as suggested by one of the Reviewers. The paper now ends on Revised Figure 5, now Figure 7.

-Revised Figure 5 (now 7): The measurements of the RHO-1 biosensor, and GFP::WAVE (in place of a Rac1 biosensor), were previously of the pharynx or nerve ring. These measurements were replaced by epidermal comparisons. Measuring those in the same movies showed significant differences for the CED-12 “GAP” mutant.

-The *ced-5* CRISPR mutation made to interfere with GEF function was re-numbered and relabeled. Allele number *pj76* was used previously for another strain (mKate2::WVE-1), therefore, this mutant is now *ced-5(pj81).* As part of toning down the claims, we refer to this mutant now as the *ced-5(pj81)* Rac1/CED-10 Binding mutant, not a GEF mutant, since this mutation seems to do more than remove GEF function.

Other helpful comments to fix typos were incorporated.

Detailed responses to each comment are included below.

Looking forward to your response.

Sincerely,

Martha Soto

Responses to Reviewers' Questions

Reviewer #1: The study from the Soto lab reported distinct roles of CED-5/CED-12 (DOCK/ELMO) in actin assembly during apoptotic cell clearance and embryonic cell migration. It has been known that two component GEF, CED-5/CED-12 functions as the GEF of CED-10/Rac1 during apoptotic cell engulfment; however, their potential roles in cell migration remains unclear. This study reported that CED-5/CED-12 (DOCK/ELMO) plays roles in embryonic cell migration, which is not a surprising results. However, they found that the loss of CED-5/CED-12 led to an increased level of F-actin in the leading edge and the membrane becomes more dynamic (data quality comments below). The author suggests that CED-5/CED-12 may inhibit actin assembly in the migrating epidermis. Further, they did some residue replacement experiments to suggest that CED-12 may act as GAP. Their data further suggests that other small GTPase such as RHO-1 and CDC-42 might be involved. While it is possible that CED-5/CED-12 may indeed play distinct roles of different events, they need obtain more solid evidence to support their major conclusion. Alternatively, they can tone down their conclusions.

> **Response:** We have toned down the conclusions in several places, including the title and the Abstract, and added new figures in support of our main findings.
>
> RE: "not a surprising result:" Two of the original discoverers of CED-12/ELMO, Drs. Zheng Zhou and Tina Gumienny, heard a presentation on this data in 2022 and were quite surprised. They had not noticed that 9-14% of the embryos die during morphogenesis. Therefore, they had missed the fact that CED-12 has an epidermal cell migration role.

First of all, visualizing F-act amount is not very trivial. Although different markers have been established, the field also pointed out various caveats using these marker. Usually, if the established marker reveals a finding that is consistent with the previous reports, it can be fine. Otherwise, a second marker should be used to backup their conclusion. In this scenario, the author can use the fluorescence phallodin to re-examine the upregulation of F-actin amount. This is a crucial experiment and should be easily performed in C. elegans embryos.

> **Response:** We were skeptical at first too. Therefore, the surprising main result in this paper, that loss of *ced-5* or *ced-12* leads to increased F-actin in the embryonic epidermis, has been validated independently by several lab members, using multiple F-actin reporters. We can do phalloidin staining, but live imaging is more accurate and reproduceable. Phalloidin in our experience gives more noise, since fixation can create differences in samples. We have measured the effects using several transgenes: besides *Plin-26::Lifeact::mCherry* we had previously crossed the *ced-5(n1812)* mutation into *ced-10::gfp, arp-2::gfp* and *gfp::wve-1,* and all of these appeared elevated in the *ced-5(n1812)* mutant.

We added an new Figure, Fig. 2A,B. The increased signal of *ced-10::gfp* is particularly striking.

Following the above point, the Figure 1E is not very supportive. How did the author measure the leading cell dynamics? Is the leading cell or the leading edge of a migrating cell? It seems that there is a small increase; however, it is indeed quite subtle. Can the author measure how much increase? How does this level increase correlate with increased F-actin amount? The author should present image frames from time-lapse movies to show the dynamics.

Response: We have previously published on the F-actin dynamics of these cells. For example, Bernandskaya et al., 2012, Figure 3. We agree the description (and labeling) for Fig. 1E could be improved. Therefore we have added a separate Figure, new Figure 2C, showing the leading edge dynamics and also showing larger frames from time-lapse movies.

Considering that fluorescence Arp-2/3 complex, WSP-1 and WAVE have already been deposited in the CGC, the author should examine if these actin nucleation factor also upregulated in the leading edge.

Response: This was added to Fig. 2A, B. *ced-5(n1812)* crossed into CED-10::GFP and GFP::WVE-1. We had old movies of *ced-5(n1812)* crossed into ARP-2::GFP, but the signal was just too low to be convincing.

While not very necessary but definitely helpful, the biochemical analysis of the GEF or GAP activity should be presented or discussed.

Response: We added in the Discussion, page 13,
"To further investigate which GTPases are targets of the proposed GEF and GAP functions of CED-5/CED-12, it may be possible to test binding of the purified CED-5/CED-12 complex to GTP and GDP loaded versions of the three main GTPases, as we and others have done (Neukomm et al., 2011, Wallace et al, 2018). This will require working out the conditions for purifying the active complex….."

The title is quite confusion: can the author specify when promote and when inhibit? If promotion is known, the author can emphasize the novel findings.

Response: We are not sure what the suggestion is. Does the Reviewer want us to just emphasize the inhibition? We prefer to mention both, since we do not assume everyone knows what DOCK/ELMO does to F-actin levels. The data in our manuscript are clear: for corpse engulfment, loss of CED-5/CED-12 leads to less F-actin, while for ventral enclosure it leads to more F-actin (and this happens in the same cells).

The writing is too lengthy. Many descriptions in the introduction have already been in the textbook.

Response:
A large effort was made in the Introduction to connect two fields that had not been connected before, branched F-actin regulation for corpse engulfment and for epidermal morphogenesis. We tried to trim the content, after adding all the suggested changes, and trimmed the text on pages: 2, 34 and 11. Please let us know about this textbook you mention. I will order it for my students.

The author should follow C. elegans gene/ protein nomenclature: it is CED-5.

Response: We call it CED-5, and are not sure where we may have been mistaken.

Reviewer #2:
The small GTPase Rac/CED-10 is known to be required for ventral epidermal cell migration during embryonic morphogenesis, but the Guanine Nucleotide Exchange Factor (GEF) activating it has not been characterized so far. The authors ask whether the bipartite Rac-GEF CED-5/CED-12 could fulfil this role. They find that strong mutations in those proteins affect the speed of ventral enclosure and lead to an increase in actin dynamics at the leading edge, arguing that they do present the hallmarks of proteins being required for ventral enclosure. However, those ced-5 or ced-12 mutations do not enhance the lethality of a partial ced-10 mutation. In search for an explanation for this conundrum, they remark that CED-12 presents some discreet homology to the active site of Rho GTPase Activating Proteins (RhoGAP). They find that mutating a critical Arg residue in this putative CED-12 GAP motif behaves as a partial suppressor of the RhoA effector Rho-kinase/LET-502, and that null mutations in ced-5 or ced-12 enhance a let-502 hypomorph. Likewise, the CED-12 GAP mutation suppresses a mutation in the Cdc42 effector WSP-1, suggesting that CED-5/CED-12 might act through Rho or Cdc42. Finally, they find that the mutation in the putative CED-12 GAP domain enhances actin level at the leading edge of epidermal cells during ventral enclosure, whereas a mutation in CED-5 within a domain with partial homology to the Rac-binding domain of the CED-5 vertebrate homolog DOCK2 removes actin from the leading edge.
They propose that CED-5/CED-12 would act as a bona fide RACGEF in apoptosis, but not during ventral enclosure. Instead, they argue that it acts as a GAP for Cdc42 and Rho to limit the amount of linear actin.

The genetic results are in general compelling, although the differences are sometimes limited such as the enhancement of wsp-1(gm324) lethality by ced-5/12, or only double lethality such as the effect on let-502(sb118), both of which are slightly indirect and the change in lethality might be significant only at another stage than ventral enclosure; the interactions with Rho-1(RNAi) or Cdc42(RNAi) are also contradictory as to the role of CED-5 vs CED-12 on RhoA. So, I take positively their genetic arguments, but I am not yet convinced by the broader conclusions. The authors argue that the putative CED-12 GAP domain is unlikely to come in contact with Rac/CED-10 owing to its position in the structure. Given the homology between Rac, Cdc42 and RhoA, how would CED-5/CED-12 be more prone to regulated Cdc42 and RhoA? Additionally, isn't it surprising if the putative CED-5/12 GAP domain regulates filopodia inducing GTPases that there aren't filopodia visible during ventral enclosure?

Response: These comments by the Reviewer were extremely helpful for revising the manustcript. We have tried to address these all. To respond to each of these points:

a. *Change in lethality might be significant only at another stage*: The Reviewer raises an excellent point that is easy to address, as we have explained in our previous work (for example, Bernadskaya et al., 2012). We typically compare three categories of dying embryos: Early arrest (no differentiation), Ventral Enclosure arrest (tissue differentiate, but no epidermal migration = Gex phenotype, Soto et al., 2001); Elongation arrest (epidermis encloses but elongation fails). These categories were scored by the first student to work on this project, author Sushma Mannimala. We have added her data to Table 1. We also add the causes for lethality throughout the text.

It is true that the molecular interpretation of these changes is open to interpretation. That is why we also tried to measure effects on the RHO-1 and CDC-42 biosensors.

b. *Given the homology between Rac, Cdc42 and RhoA, how would CED-5/CED-12 be more prone to regulate(d) Cdc42 and RhoA?*
This important question involves two distinct points.
First, it is not fully understood how GEFs can distinguish between different GTPases. However, to date, DOCK GEFs are known to act as GEFs on Rac and CDC-42 and never on RHO-1. Structural studies provide arguments to explain this (Kulkarni et al., 2011 DOI 10.1074/jbc.M111.236455; Reviewed in Boland et al., 2023 doi: 10.1002/1873-3468.14523).
Second, the structure shows that in the open and closed conformation the GAP domain faces away from the Rac binding domain and is not buried in other regions. This simply says it is available to act as a GAP in either conformation. Revised Figure 4 (Fig. 5) adds a light blue "membrane" and the membrane attachment by the DHR1 AAs to help orient readers about how the closed complex is proposed to attach to the membrane.
Finally, the evidence we provide that the GAP domain may act on RHO-1 or CDC-42 is functional (based on the genetics and the imaging), not structural. Genetics experiments strongly suggest the GAP function affects both RHO-1 and CDC-42 pathways (Revised Figure 7B). We have changed the Title and Abstract to show that interactions with RHO-1 and CDC-42 are supported but not proven by our data.

c.*Why don't we see filopodia?*
We never say that there are no filopodia, but agree the images did not illustrate the protrusions well. We have added images of close ups of the cells to illustrate the protrusions [Fig. 2C]. In fact, we use the term "protrusions" which can include lamellipodia and filopodia (added, page 4, "We use the term "protrusion" here and throughout, since the leading cells make membrane extensions that encompass both lamellipodia and filopodia.") Imaging F-actin dynamics shows that all of the cells, the leading cells and the pocket cells, make sharp thin protrusions that may be filopodia (Fig. 2C). When we removed *arp-2* by RNAi, thus strongly reducing branched actin, we see all of the cells making thin protrusions that may be called filopodia (Patel et al., 2008), presumably because formins are still active. So, to answer this query: yes, CED-5/12 GAP domain may be regulating protrusions that include filopodia.

Major points before the work can be accepted:
1. The authors should model Cdc42 and RhoA with CED-5/12 as they did in Fig. 4 with Rac.

> **Response:** This was added to Supplemental Fig. S1C. The key residue of the GTPases that needs to bind to the GEF for stability and catalysis is CED-10/Rac1 W56; RhoA/RHO-1 W58; CDC-42/Cdc42 F56. For the GEFs CED-5(S1541/R1542) or DOCK2, (S1528/M1529), only CED-10/Rac1 W56 makes the correct contacts, based on the distances and the orientation. In Supplemental Fig. S1C we added an image of all three GTPases trying to bind to CED-5/DOCK2, showing incorrect distances and orientations for CDC-42 F56 and RHO-1 W58, while Rac1/CED-5 W56 fits the published structures.

2. They should make sure that the enhancing or suppression lethality effects described in Fig. 5 are due to ventral enclosure defects, rather than any other embryonic defect involving the two players being tested.

> **Response:** We agree this is a complication, and decided to take out the cdc-42 and rho-1 RNAi. However, we repeated the RNAi of *cdc-42* and *rho-1* to examine the categories. *cdc-42* RNAi led to mainly ventral enclosure lethality (2/3 of the deaths) with the rest from

elongation arrest (1/3 of the deaths). *rho-1* RNAi similarly led to ventral enclosure and elongation arrests.
The *rho-1* pathway was removed using the RHO Kinase mutation, *let-502(sb118ts)*. Under the conditions used, the embryos mainly died during elongation, and the double mutants also showed this. *wsp-1* mutants mainly die during ventral enclosure, with a smaller number dying during elongation, and the double mutants also showed this.

3. They should explain why there is no apparent filopodia visible in Fig. 6A. If CED-5/CED-12 acts in pocket cells in the second step of ventral enclosure, which involves non-muscle myosin II and presumably RhoA/Rhokinase, then they should demonstrate changes in the level of actomyosin in those cells.

Response: We added Fig. 2 to address this. All of the embryos show protrusions that include filipodial changes. Here we focus on leading cell/ leading edge F-actin levels.
Ventral enclosure depends on the 4 leading cells initiating the migration (Williams Masson et al., 1998, https://doi.org/10.1006/dbio.1998.9048). Protrusions also occur in more posterior cells. Excess RHO-1 could interfere with the migrations in both the leading cells and the pocket cells. However, if the leading cells are not able to correctly initiate and continue the migration, it really does not matter what is happening in the pocket cells. The migrations will arrest (Williams-Masson et al., 1998).

4. It was not entirely clear to me what is the explanation for the fact that both ced-12(pj74)-GAP and ced-5(pj76)-GEF both increase RhoA in the pharynx as evidenced from the ANI-1(AH-PH) marker.

Response: We made new measurements focused on epidermal cells and removed the pharynx measurements. Epidermal seam cell measurements show that the ced-12(pj74)-GAP increased RhoA in the epidermis, but the *ced-5(pj81)* Rac1 binding mutation did not change the levels significantly. This revised Fig. 5 is now Fig. 7D.

Minor:
1. Figure 2B-D: the median value in grey is difficult to spot; please consider another colour.
Response: We changed the lines to blue and it looks much better, thanks.

2. Figure 3A-B: my eye looking at the ced-5 and gex-3 RNAi images of actin is disagreeing with the quantification displayed in panel B, inasmuch I see lower actin signal for ced-5(n1812) compared to gex-3. My eye may be fooled, but perhaps the example for ced-5 or gex-3 does not reflect the average. Please revisit.

Response: Yes, we agree the movies chosen do not match the measurements shown. A more representative *ced-5* movie was added. We added details to how the measurements were done, checked the measurements on the movies used.

3. Page 7, line 19: syntax problem for “and did loss” which is lacking a subject or some other word.
Response: Thank you. Changed to “as did loss”

4. Page 9, line 4/5: it is let-502(sb118), not sb1008.
Response: Thank you. Fixed.

5. Please display the strain list in a separate Table, rather than a text. It will make it more readable.
Response: We have done this.

Reviewer #3:
Knowing that CED-12 and its partner CED-5 function as a GEF for CED-10/Rac1 to promote corpse engulfment of dying cells during embryogenesis and distal tip cell migration in C. elegans, Venkatachalam et al. characterize the role of CED-5/CED-12 during embryonic epidermal cell migration. The authors found that inhibition of CED-5/CED-12 led to some embryonic lethality but, counterintuitively, resulted in increased F-actin levels and cell migration velocity. To address how CED-12/CED-5 could inhibit F-actin in this cellular context, they investigated the role of a GAP domain that has not been described previously. Mutating a candidate catalytic Arginine in the CED-12 GAP region (conserved and usually mutated to inhibit other GAPs) resulted in decreased embryonic lethality when compared to the null cdc12 mutant, and specifically increased F-actin levels in epidermal migrating cells but not in engulfing corpses. Mutating a candidate GEF region on CED-5 predicted to bind and stabilize Rac1 for GTP transfer, resulted in apparent failure in both corpse engulfment and epidermal cell migration. By doing genetic experiments and assessing levels of reporters for 3 GTPases, the authors propose a model where CED-5/CED-12 function both as a GEF and a GAP, supporting the cycling of multiple GTPases that may vary depending on the cellular context.

Although the main findings are interesting and novel, the manuscript and experimental work therein could be more rigorous, more thorough and better explained. It is my opinion that the manuscript should be significantly improved before considering it for publication in PLoS Genetics. Below follows a list of comments/suggestions for improvement.

Major comments:

The mutants that were generated were based on predictions. What validates that they really correspond to a CED-12 GAP mutant and a CED5 GEF mutant?

> **Response:** The validation presented in this study is functional, not biochemical. The GAP mutation is doing precisely what one would expect: rescuing partial loss of the GTPase. The GEF mutation, as we explain in the text, likely interferes with more than GEF function so it has been renamed Rac binding mutant, and less emphasis is made on its interactions with the three GTPases. There is no simple amino acid change that has been proposed to cleanly mutate GEF function, unlike the well supported use of mutating the catalytic Arginine (R) to block GAP function.
>
> To mutate the GEF function, we tried mutating the PPP motif that suggested was needed for GEF function (Gumienny et al., 2001; Lu et al., 2005), and got a mild, partial phenotype. More recent work suggests the PPP is not essential for GEF function. We used the new structures to design a mutation that would interfere with GEF function. Blocking Rac1 binding seemed a good way to interfere with GEF function. However, as we clarify in the revised draft, page 9, this mutant is different from simple loss of function, since it reduced epidermal F-actin, the opposite of the ced-5 null. We added on page 9, “We refer to this mutant as the *ced-5(pj81)* Rac1/CED-10 Binding mutant.”

An immunoblot to show protein levels in the several mutants could be shown. If the mutated protein is not expressed at wild-type levels, care has to be taken when taking conclusions.

> **Response:** We do not have antibodies to do this experiment.

Figure 5B: Explain to the reader why would the results obtained with a GAP null be different from a GAP point mutant.

Response: We assume the Reviewer meant why a null mutation in *ced-5* or *ced-12* is different from the *ced-12* GAP mutant? We say on page 10, "These results suggested the GAP activity of CED-12 acts on RHO-1, while the GEF function acts in parallel to RHO-1."

Figure 5B: Should add the GEF point mutant (ced5(pj76)) in addition to the ced5 null.

> **Response:**
> We did not pursue this since we are no longer calling it the GEF mutant. The results would be hard to interpret.

Figure 5C: Effect of AHPH Rho biosensor should be assessed during epidermal cell migration - why looking at pharynx and buccal activity?

> **Response:** Agree. We made this change in revised Fig 5C, now 7D.

Effect of cdc42 biosensor should be assessed during epidermal cell migration
Measurements with GFP::WVE-1 - increase N and assess during epidermal cell migration

> **Response:** We added measurements of epidermal cells. For CDC-42 we measured epidermal cells, just as migration is set to start. For GFP::WVE-1, which is very faint, we show epidermal seam cells in 1.5 fold embryos. The migrating epidermal cells from the ventral view are quite thin, so measuring their levels and not measuring the signal underneath them was problematic. The epidermal signal we show, and the fact that the signal is either decreased (*ced-12* GAP) or unchanged (*ced-5* Rac binding mutant), supports the data presented. The drop in *gfp::wve-1* for ced-12 GAP mutant was seen in all tissues measured. We try to address this result in the Discussion.

Opposite messages: In the text it says that "…while depleting rho-1 in ced-12(pj74) GAP mutant reduced lethality to 8%, and this drop was significant (Table 3)." In table 3: ced-12(pj74); rho-1 RNAi 0.76 lethality"

> **Response:** Thank you, this was a typo. Table 3 should be 0.076, i.e. 0.08 = 8%. This has been fixed. Table 3 is not needed since we show the main results in Fig. 7B., but this can be included in the submitted materials.

"cross the candidate GAP to hypomorphic alleles, or partial loss of function, of the target GTPases. The prediction is that loss of a GAP in combination with a hypomorphic allele (ADD of the small GTPAse) will rescue the loss of function phenotype… loss of a GEF in combination with a hypomorphic allele (ADD of the small GTPAse) will synergistically enhance the loss of function phenotype."

> **Response:** Thank you. Fixed, bottom of page 9.

- However, loss of let-502 or wsp-1 brought embryonic lethality back to the mutants alone and did not improve it further. Explain.

Response: What page is this on?

“Neither loss of the GAP function, nor the GEF function increased the levels of GFP::WVE-1. Instead, loss of the GAP function using ced-12(pj74) resulted in significantly reduced gfp::wve-1 levels in all tissues measured, including in the nerve ring (Fig. 5E). ced-5(p76) resulted in no significant change. This result further supported that the GEF function does not simply promote active Rac, and suggested CED-12 is not a GAP for CED-10/Rac1”

-> if ced5 is a GEF for Rac1, shouldn’t WAVE signal decrease in the cdc5 GEF mutant? decreased?

Response: We do not think CED-5 is a GEF for Rac1 for the epidermal migrations, so we do not expect to see signal decrease. We re-wrote this:

“If CED-5/CED-12 act as a GEF for CED-10/Rac1, loss of the GEF function should reduce levels of GFP::WVE-1. Interfering with Rac1 binding did not significantly change the levels. If CED-5/CED-12 act as a GAP for CED-10/Rac1, loss of the GAP function should increase levels of GFP::WVE-1. The levels did not increase and instead dropped in all tissues measured, including the epidermis (Fig. 7C).” page 10

If CED-12 is not a GAP for Rac, then levels of the Rac1 reporter should not be affected; why are they decreased?

Response: We reviewed this in different tissues and at different stages, and the levels are definitely down. This shows it is not so simple to interfere with GTPases. It is possible that higher levels of another GTPase in the GAP mutant (RHO-1, CDC-42) can indirectly affect Rac1 by competing for F-actin, or complex cross-talk. We discuss this now in the Discussion, page. 13.

Add p values to all tables
Response: The Tables now include asterisks to denote P values.

Should quantify defects in cell migration velocity for the GAP mutant
Response: We did not do this due to time constraints. With higher F-actin, and faster enclosure, we predict they are higher.

ced5 GEF mutant - quantification of corpse engulfment or epidermal cell migration missing
Response: This is presented in Figure 6, B and C.

Discussion - needs to be significantly improved:

“fully penetrant increase in F-actin and partially penetrant embryonic lethality”. Embryonic lethality is rather low - Doesn’t this mean that the phenotype is not due to increased F-actin? are the embryos that die expressing more actin?

Response: This is discussed on page 11. We have added more emphasis and changed “all” to “100%”. 100% of the embryos express more F-actin. On page 11 explain that we have seen this for other embryonic mutants: 100% change in F-actin, partial lethality (Wallace et al., 2018, Added: Raduwan et al., 2020).

“definitively show that the embryonic lethality of ced-12, also seen in partner protein ced-5, is due to failure to regulate F-actin during embryonic cell migrations, resulting in increased F-actin, altered

dynamics and faster epidermal enclosure as the cells increase their migration rate" - how do the authors know that lethality is due to misregulated actin levels? increased F-actin and consequent increased velocity in migrating cells is the cause of increased lethality? this was not tested.

Response: We changed the language to "strongly support."

Also, was decreased actin in corpses a possible cause for lethality? is this published? if corpses are not engulfed, are the embryos viable?

Response: In *C. elegans* failure to engulf corpses is compatible with embryonic development. Embryos seem to tolerate the un-engulfed corpses. This may be why the embryonic morphogenesis role of these proteins, CED-5 and CED-12, has not been the focus of study.

"Mutations in the rho-1 pathway, like filamin, or the Rho GAP SPV-1 lead to embryos that are round (Kovacevic and Cram, 2010; Tan and Zaidel-Bar, 2015.), so too much or too little actomyosin contractility can alter egg shape, including producing round embryos" -> In what way does filamin affect actin levels? unclear whether the authors are talking about actomyosin in the spermatheca or in the embryos

Response: We added "in the spermatheca" to that sentence, since the work on SPV-1 is focused on the spermatheca: "…too little actomyosin contractility <u>in the spermatheca</u> can alter egg shape..."

"the same epidermal cells that showed increased F-actin in the migrating cells, also show reduced F-actin around corpses" - is it the same cells? if yes, is it that the total F-actin is normal but the distribution of F-actin within the same cell is not?

Response: Yes! It is the same cells. That is what got us interested in this. We had included this, "in the same tissue", in the original Title, but were told it was too confusing. Regarding are total F-actin levels up, or just re-distributed: very good point. Since there is such high F-actin overall in the epidermis, but the only decrease is around the corpses, it seems that levels overall are increased, except around corpses. We added Fig. 4D to illustrate this point.

"This mutant, ced-5(pj76), resembled null mutations in ced-5 and ced-12, since it altered corpse engulfment and epidermal cell migration, with similar levels of embryonic lethality. However, it had opposite effects on epidermal F-actin levels (lowering them) (Fig. 6A,B). One explanation for why mutating these two Rac1 binding residues is worse than simply deleting ced-5 is that this "GEF" mutation traps active Rac1 molecules, acting as a Rac1 sink. We have proposed that other GEFs can activate Rac1, but in animals with the ced-5(pj76) mutations, other Rac1 GEFs may lose access to Rac1. Interestingly, this mutant also led to increased active RHO-1 (Fig. 5C)." -> In what aspect is the GEF mutant worse than simply depleting CED-5? Possible explanations for increased Rho activity? Possibly mention the case of MgcRacGAP: work from Oegema, Canman and Glotzer groups.

Response: Yes, it is important to point out that our attempt to create a "GEF" mutant is not so simple. The CED-5 "GEF" mutant showed decreased CED-5 functions (embryos failed migrations to enclose epidermis and migrations to enclose corpses), but the F-actin effects are distinct from the *ced-5* or *ced-12* nulls, especially in the overall low F-actin. Also, when we repeated measurements of the "Rho biosensor" in the *ced-5*-Rac binding mutant, we did not see an

increase. Rather, Rho biosensor levels in the epidermis were not significantly changed. We have added on p. 13, “Previous studies on GTPases show that cross talk between GTPases and their regulators make the interpretation of knock down studies complicated (Bement et al., 2023). For example, loss of the RhoA GAP CYK-4/MgcRacGAP decreases RhoA function during cytokinesis, since this RhoA GAP is required to activate the RhoA GEF ECT-2 (Zhang and Glotzer 2015).”

“Therefore, while mutating the CED-12 GAP function only affected the cell migration function of CED-5/CED-12, and not the corpse engulfment function, mutating the GEF function in CED-5 affected both processes.” -> Where is it shown that CED-5/CED-12 is a GEF for Rac in epidermal cell migration? in fig 5E, WVE-1 levels do not decrease in ced 5 gef mutant.

Response: Correct. The Rac1-binding domain “GEF” mutant did not affect GFP::WVE-1 in the nerve ring, or in the epidermis. The key experiment would be to measure corpse levels of GFP::WVE-1 or CED-10 (if we had a biosensor, which we do not). GFP::WVE-1 is not easy to measure at the corpses since the levels are so low to begin with.

Model does not necessarily reflect findings, p.e the fact that branched/linear actin inhibit one another has not been documented in these cells. Also, cdc12 GAP function does not seem necessary for corpse engulfment according to Fig. 6D. [Correct.] Rho biosensor levels were not measured in the migrating epidermal cells or engulfing corpses to be able to conclude that ced5/12 is a gap for rho in both processes. The authors should provide possible reasons for why would the same protein complex be able to promote and inhibit branched actin?

Response: We modified the model slightly. Epidermal Rho biosensor was measured. The Reviewer is correct that the GAP function is not predicted to be required for corpse engulfment. That arrows were straightened to show this more clearly. We think the model helps summarize a complex story. Some predictions made by the model (branched vs. linear F-actin imaging) would require new reagents not currently available.

To address the Reviewers suggestion for reasons why, we added to the last paragraph, p. 13, “How may one protein complex be regulated to both promote and inhibit F-actin? Our model predicts that different upstream regulators recruit CED-5/CED-12 for the two processes, epidermal cell migration vs. epidermal corpse engulfment. “

“This suggests too much or too little WSP-1 has same phenotype: too much F-actin, and some dead embryos.” How could that be? add possible explanations? is the wsp1 mutant wsp-1(gm324) a loss of function allele?

Response: Yes it is a loss of function allele. This is analogous too much or too little Rho signaling has the same phenotype, in spermathecal function, as mentioned on page 11.

Minor comments:

Fig. 1B improve labels
Add LifeAct::mCherry probe. Add REF in legend.
Explain better that 2nd column in B is the magnified view of the white box in the first column
How was the position of the line as well as its beginning and end decided?
Should have a membrane marker to outline cells.

Response: Most of the above was added. Measurements were done at the brightest part of the Leading Cells, which is several microns behind the leading edge. However, the overall levels

are much higher, so any region compared, except at the corpses, gave higher measures. We have tried but do not yet have a membrane marker for just the epidermal cells.

Fig 1C: “Loss of the GTPase CED-10/Rac1, showed significantly reduced levels of F-actin in leading cells, and arrested migrations“
ced10 mutant - only one leading cell is observed - probably because of enclosure failure but please mention that.

Response: We added a comment on page 4.”Only one leading cell is visible in the ced-10(tm597) embryo due to the enclosure failure and the angle of the movie.” We also added a dotted line to show the entire embryo, including the regions that are not enclosed.

Fig. 1E - maybe stills of a video should be provided for one to have a better perception of what was considered a protrusion and how protrusions were measured. Please provide details, none are provided in the methods section. In movie S2, the leading front of migrating cells seems dynamics in all situations.

Response: We added Fig. 2 to address this.

“We obtained the same results with multiple null or strong loss of function alleles, including ced-5(n1812, n2002),ced-12(n3261, ky149), and when ced-5 was removed by RNAi.”
Shouldn’t these data be shown in supplement?

Response: We added some of the mutants to revised Table 1.
We did not have good counts of n2002 so it was removed.

1F: unclear - why only a row of cells is observed in 2nd, 3rd and 5th cases? panel not effective - better to clarify what failure of migration should look like (mention movie S1), unclear what Gex phenotype should look like - a schematic should be added. Also, embryonic lethality is not so high, so the images shown probably do not reflect the most representative cases

Response: Fig. 1A is expanded to better explain the normal development, and what happens to arrested embryos. We revised Fig. 1F, now Fig. 1E, added dotted lines to show outline of the un-enclosed embryos, and moved the arrows to emphasize where the migration arrest occurs, added % of embryos showing phenotype to address concern of “how representative”. Figure legend added that these are age matched, i.e. at the time when WT has enclosed, these mutants have arrested. Together with revised Fig. 1A, this should help people appreciate the phenotype.

Quantitative analysis of Gex phenotype should be added to table 1, to make it easier to understand whether embryonic lethality could be due to other phenotypes.

Response: Table 1 was revised to reflect the 3 stages of arrest we monitored. These match the schematic in Figure 1A.

Correct this sentence: “Proper migration of the embryonic epidermis requires dynamic F-actin at the leading edge, that is and ventrally enriched (Bernadskaya et al., 2012).”

Response: Thank you. We cut the “and”.

Figure 2: could be moved to supplement

Response: We prefer to keep it in the main text. This is the first report documenting that branched actin mutants have this phenotype. Moving it to the supplement means it will not be seen by most. This is the first paper documenting the important role of CED-5 and CED-12 in embryonic morphogenesis. The cell death community had missed these embryonic phenotypes.

3A: Label C1, C2 and C3 corpses because all of them will be used for quantitative analysis in 3B.

Response: We added a cartoon to show C1, C2, and C3, Fig. 4B. It is hard to image all 3 at once, so the images in Fig. 4A, of one Z slice, show C1. In the new panel, Fig. 4D, which zooms in on the left side, C1 and C3 are sometimes visible.

3A, B: quantification of LifeAct in B does not match images in A, where LifeAct is more notoriously decreased in ced5 mutant - are they not representative?

Response: We went through the movies, checked measurements, and replaced the *ced-5* mutant with one that is more representative.

Correct this sentence: "Loss of gex-3, an effector of CED-10 with cell migration and corpse engulfment defects (Soto et al., 2002), resulted in reduced F-actin levels around the corpses, and did loss of ced-5(n1812), or ced-12(n3261)."

Response: Yes, two reviewers caught this. Changed to "as did loss"

3C: confusing. Maybe split schematic in 2 rows, one for corpse engulfment (where CED5/CED12 promote Rac activation) and another for cell migration (where CED5/CED12 inhibit Rac activation) - like in 5A

Response: We added Fig. 4D so emphasize that two different things are happening in those epidermal cells. We hope this helps.

"of alternative forms of CED-5 or CED-12…current molecular model for CED-5 predicts a single protein, and the model for CED-12 predicts two proteins that differ only by 7 amino acids at the N terminus - model? proteins should be isoforms?

Response: The data are based on bioinformatic predictions, listed on Wormbase. We are not aware of any experimental evidence for the two isoforms. It would be hard to show by western blot, since the size difference is 7 aa.

4A: alignment with ELMODs is not great and worse alignment with ELMOS is not shown - comment on first and add second

Response: We added alignment of the CED-12 GAP region with 3 human ELMOs (Fig. S1A). Since we show this next to CED-12 vs. worm GAPs alignment, it is even more striking that ELMOs have this "GAP"-like region. To keep the Figure small, we only include part of the alignments around the putative GAP regions. In Fig. 4A, we added a cartoon of the proteins, with main motifs, location of the "GAP" regions. We hope helps people visualize first the proteins, then the motifs, then the structure.

4B-D: Arginine 537 does not seem to face out in 4C. unclear how Arginine 537 faces away from the membrane in the closed configuration - where is the plasma membrane in 4D?

Response: To orient readers, we added a shaded blue region to show the membrane, and emphasized the amino acids that insert into the membrane by using "ball" representation so they stand out more in Fig. 4 (now Fig. 5) In the text, page 8 we added, "Since R537 is not buried in a pocket in the open or closed conformation, it may be open to interactions with other proteins."

Improve sentence - "The aligned structures showed that the catalytic Arginine of the proposed CED-12 GAP, R537 faces out, away from DOCK/CED-5 and Rac1/CED-10, and away from the membrane, in the DOCK2/ELMO1 structure in both the open and closed conformations"

Response: The sentence was re-written as:

"The aligned structures showed that the proposed CED-12 GAP's catalytic Arginine, R537, faces away from the membrane, and away from DOCK/CED-5 and Rac1/CED-10. This is true for CED-12 R537 aligned to the open and closed conformations of DOCK2/ELMO1."

What is the point of this sentence: "Human ELMO has Q541 in place of R527 (Fig. 4B)"?

Response: This was reworded, "Human ELMO does not have R537, and has Q541 instead." We are trying to emphasize that we can thread the two structures and compare which residues are where. Please see also Suppl Fig. 1.

Spelling mistake: "…consulted the DOCK5/ELMO1 structure…"

Response: Not sure what the mistake is. This is supposed to be DOCK5/ELMO1.

Add mention to Fig. 4B in this sentence: "M1529 of DOCK2 is replaced in CED-5 by R1542. Using UCSF Chimera (Pettersen et al, 2004; Goddard et al., 2018), we predicted that R1542 would make similar hydrogen bonds with Rac W56/CED-10 W56."

Response: Added (Fig. 4B), now (Fig. 5B).

"compared to 14-16% for two putative null alleles of ced-12 (Table 1, Fig. 5B, Movie 2)." Add names of null alleles of ced-12

Response: Added, "*ced-5(n1812)* or *ced-12(n3261, ky149)* null alleles", page 8.

"We predicted that loss of GAP function would suppress partial loss of a GTPase." repetition

Response: The sentence was cut.

"Loss of the proposed GEF function, using ced-5(p76) resulted in even more elevated active RHO-1. This result supported the genetic result that the GAP function regulates RHO-1."
-> it should be "This result indicated that ced5 is not a GEF for Rho."

Response: Thank you. Fixed.

Spelling mistake: "…ced-12(n32561 null)"

Response: Thank you. Fixed. changed to: "*ced-12(n3261-null)*"

Figure 6 could come before figure 5.

Response: We made this change. This order makes it clear right away, based on F-actin levels, that the ced-5 Rac1 binding mutant likely interferes with more than GEF fuciton.

Discussion: "Since the effect" - add effect of what

Response: Thank you. Fixed. "Since the effect of mutating the CED-12 GAP function"

Model in Figure 5E, mentioned in the discussion, should be corrected to 6E.

Response: Thank you. Fixed.

Have all data underlying the figures and results presented in the manuscript been provided?
Large-scale datasets should be made available via a public repository as described in the *PLOS Genetics* data availability policy, and numerical data that underlies graphs or summary statistics should be provided in spreadsheet form as supporting information.
Reviewer #1: Yes
Reviewer #2: Yes
Reviewer #3: Yes

PLOS authors have the option to publish the peer review history of their article (what does this mean?). If published, this will include your full peer review and any attached files.

If you choose "no", your identity will remain anonymous but your review may still be made public.

Do you want your identity to be public for this peer review? For information about this choice, including consent withdrawal, please see our Privacy Policy.
Reviewer #1: No
Reviewer #2: No
Reviewer #3: No

In compliance with data protection regulations, you may request that we remove your personal registration details at any time. (Remove my information/details). Please contact the publication office if you have any questions.
